# Beyond the Surface: Uncovering Secondary Causes of Osteoporosis for Optimal Management

**DOI:** 10.3390/biomedicines12112558

**Published:** 2024-11-08

**Authors:** Rasheed Hosein-Woodley, Rahim Hirani, Ali Issani, Anum S. Hussaini, Olivia Stala, Abbas Smiley, Mill Etienne, Raj K. Tiwari

**Affiliations:** 1 School of Medicine, New York Medical College, Valhalla, NY 10595, USArhirani2@student.nymc.edu (R.H.);; 2 Graduate School of Biomedical Sciences, New York Medical College, Valhalla, NY 10595, USA; 3 Department of Orthopaedic Surgery, Keck School of Medicine of USC, Los Angeles, CA 90033, USA; 4 Department of Global Health and Population, Harvard T.H Chan School of Public Health, Boston, MA 02115, USA; 5 School of Medicine and Dentistry, University of Rochester, Rochester, NY 14642, USA

**Keywords:** secondary osteoporosis, fragility fractures, bone mineral density, treatment of osteoporosis

## Abstract

Osteoporosis (OP), a condition marked by reduced bone mineral density and increased fracture risk, can arise either as a primary disorder or secondary to other diseases and medications. While primary OP typically relates to age-related or postmenopausal changes, secondary OP results from underlying conditions or drug exposures, complicating diagnosis and management. This review explores the pathophysiology, prevalence, and treatment approaches for secondary OP arising from endocrine, renal, gastrointestinal, hematological, and autoimmune disorders, as well as medication side effects. The findings highlight that secondary OP is frequently undiagnosed, particularly in premenopausal women and men, with conditions such as chronic kidney disease, glucocorticoid use, and diabetes among the primary contributors. Management strategies must be tailored to address the underlying conditions to effectively reduce fracture risk and improve outcomes. Ultimately, this review underscores the necessity for increased clinical awareness and more targeted interventions for optimal management of secondary OP.

## 1. Introduction

Osteoporosis (OP), both an underdiagnosed and undertreated disease, is well defined by the World Health Organization (WHO) as a disorder that has a bone mineral density (BMD) T-score of −2.5 or lower, as measured by the dual-emission X-ray absorptiometry (DEXA) scan at the hip, lumbar spine, or femoral neck [Table 1] [1,2,3]. It is characterized by reduced bone mass, microarchitectural worsening of bony tissue, and decreased bone strength and is now considered the most prevalent disorder of the bone in the world [3]. To provide a diagnostic context for OP, we outline the standard levels of relevant biochemical markers and diagnostic features associated with the disease. BMD measured through DEXA remains the diagnostic gold standard, with a T-score of −2.5 or lower indicating OP, as per WHO guidelines. Normal ranges for associated biochemical markers include serum calcium (8.5–10.2 mg/dL), phosphorus (2.5–4.5 mg/dL), parathyroid hormone (PTH, 10–65 pg/mL), and vitamin D (>20 ng/mL). These parameters serve as a baseline for identifying deviations typical in secondary OP, thus enhancing diagnostic accuracy and guiding appropriate management. In the United States, 10 million individuals over the age of 50 years—8 million women and 2 million men—are reported to already have the disease [4]. In Europe, the prevalence of osteoporotic fracture in 2000 was estimated at 3.79 million [5]. In 2010, 24,000 cases of osteoporotic hip fractures among individuals over 50 years of age were reported in Turkey, a number that is expected to increase from 25% to 40% by 2050 [6]. Tarride et al. (2012) estimated more than 50,000 Canadians were hospitalized for fractures because of OP [7]. According to the data, the annual cost in the United States of America required for the care of fractures occurring secondary to OP equals or, at times, exceeds the annual cost required for the care of a patient with myocardial infarction, breast cancer, and/or cerebrovascular accidents [1].

Bone mineral density has been the accepted standard of screening for this disease and is routinely evaluated by general physicians in postmenopausal women [8]. Bone loss resulting from other disorders or medication exposures is referred to as secondary OP and is most common in premenopausal women and in men with OP, with the reported prevalence in men as high as 64%. Around 30% of postmenopausal women are also reported to have underlying disorders that may have added to their bone loss [9]. These secondary causes that are often overlooked and not considered in patients diagnosed with OP can be related to endocrine, hematologic, gastrointestinal, and rheumatologic diseases, as well as drugs and other factors [Table 2] [9,10,11]. It is important to rule out secondary causes of OP as the management of these patients may vary, and the response to treatment may be inadequate if the underlying condition is unrecognized and not being addressed [10]. We therefore aim to perform a review addressing secondary causes of OP and will provide a framework regarding their treatment.

This narrative review was conducted through a comprehensive literature search in databases such as PubMed and Google Scholar. The search utilized keywords related to osteoporosis, secondary causes, and associated conditions. Inclusion criteria encompassed studies that provided insights into the pathophysiology, prevalence, and treatment of secondary osteoporosis due to endocrine, renal, gastrointestinal, hematological, and autoimmune disorders, as well as medication-related causes. Exclusion criteria included articles that did not address secondary osteoporosis or were not peer-reviewed. The relevant literature was reviewed, and findings were synthesized to identify common themes, challenges in diagnosis, and management strategies for secondary osteoporosis.

## 2. Mechanism of Osteoporosis

**Figure 1:**
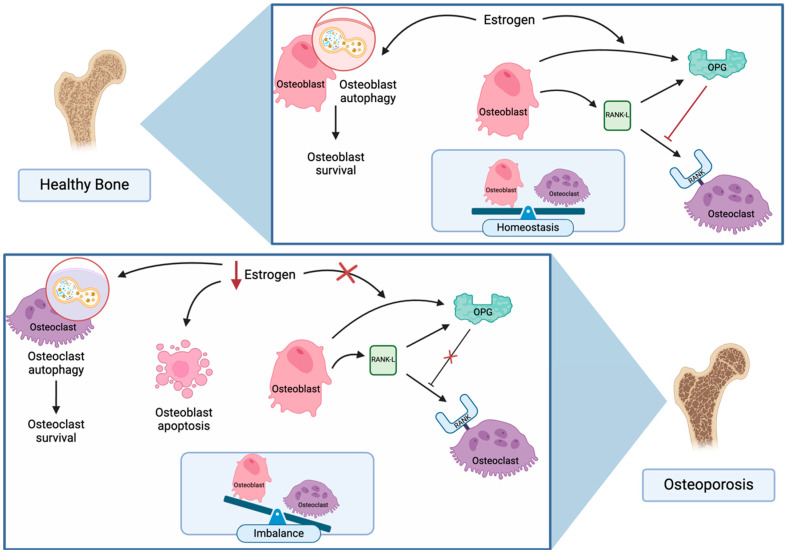
**Role of estrogen deficiency in osteoporosis pathophysiology**: A simplified graphical illustration of the role of estrogen deficiency in OP pathophysiology. Estrogen deficiency amplifies osteoclast activity via reduced apoptosis and elevated autophagy, while inducing apoptosis in osteoblasts by hindering autophagy, collectively accelerating bone loss. Osteoblasts express RANKL, which when bound to the RANK receptor expressed by osteoclasts stimulates osteoclast-mediated bone resorption. Osteoblasts also express OPG, a soluble protein mimicking the RANK receptor which can bind to RANK-L. In the presence of estrogen, OPG activity is upregulated which competitively inhibits RANK activation and inhibits osteoclast-mediated bone resorption. Estrogen deficiency reduces OPG activity, which increases RANK activation and results in osteoclastic bone resorption surpassing osteoblast-mediated bone formation. (Created in BioRender: BioRender.com/i50p644). OPG: osteoprotegerin, RANKL: nuclear factor κ-B ligand, RANK: nuclear factor κ-B.

OP occurs due to an imbalance in the bone remodeling mechanism where osteoclast-mediated bone resorptive activity surpasses osteoblast-mediated bone formation resulting in fragility fractures [12] (Figure 1). In this complex process, osteoblasts express both the receptor activator of nuclear factor κ-B ligand (RANK-L) and osteoprotegerin (OPG); and osteoclasts express the receptor for RANK-L. RANK-L binds to both the nuclear factor κ-B (RANK) receptor and OPG. Conversely, OPG, a soluble protein, acts as a decoy receptor and functions to inhibit RANK stimulation and consequently decreases osteoclastic activity and subsequent bone resorption.

The regulation of bone metabolism is significantly influenced by estrogen, which upregulates OPG activity on osteoblasts and inhibits osteoclast-mediated apoptosis [12,13]. Estrogen deficiency can result from various causes, including ovarian failure in postmenopausal women, individuals undergoing gender-affirming hormone treatment, and other conditions that lead to reduced estrogen levels or blockage of estrogen receptors. This deficiency leads to increased bone turnover and alterations in bone metabolism, impacting the concentrations of T lymphocytes, B lymphocytes, monocytes, and cytokines. The culmination of these changes leads to heightened loss of bone mineral density (BMD), disruption of trabecular microstructure, and an elevated risk of fractures.

Importantly, OP primarily involves aberrant osteoclast activation triggered by changes in apoptosis, inflammatory responses, and autophagy [14]. It is important to differentiate between primary and secondary OP, as their underlying mechanisms exhibit distinct characteristics. Primary OP, such as postmenopausal OP, primarily results from estrogen deficiency, which increases osteoclast survival and activity, thereby accelerating bone resorption and disrupting the balance with bone formation. This process predominantly affects trabecular bone microstructure, leading to heightened resorption [15]. In contrast, secondary OP, such as glucocorticoid-induced OP, exhibits distinct pathophysiological and histological characteristics. Glucocorticoids induce apoptosis of osteoblasts and osteocytes, suppressing bone formation and causing trabecular thinning with reduced connectivity [16]. Additionally, glucocorticoids increase RANK-L expression and reduce osteoprotegerin, thus promoting osteoclastogenesis through decreased bone formation rather than the increased resorption seen in postmenopausal OP [17]. The pathophysiology of secondary osteoporosis involves an imbalance in bone remodeling processes, primarily driven by underlying medical conditions or medications. Increased bone resorption often occurs due to elevated levels of hormones such as parathyroid hormone in hyperparathyroidism or cortisol in Cushing’s syndrome, leading to enhanced osteoclast activity [18]. Concurrently, factors like glucocorticoid use can suppress osteoblast function, reducing bone formation. Nutritional deficiencies, such as insufficient vitamin D or calcium, impair mineralization, while chronic diseases like kidney disease disrupt calcium and phosphorus homeostasis, further compromising bone health [19]. Inflammatory mediators from autoimmune disorders can also stimulate osteoclastogenesis, exacerbating bone loss. This complex interplay ultimately results in decreased bone mineral density and an increased risk of fractures. A detailed overview of the pathophysiology of secondary osteoporosis based on their underlying etiologies is described further in their respective sections.

In examining the role of apoptosis, it signifies a cell death process initiated by preexisting intracellular mechanisms. Aberrations in apoptosis can contribute to bone-related ailments, given that bone mass density is contingent on the lifespan changes of osteoblasts and osteoclasts through apoptosis. Concerning inflammatory reactions, pro-inflammatory signals influence mesenchymal stem cells and osteoclast precursors, expediting osteoclast-mediated bone resorption. Elevated levels of serum inflammatory markers in adults correlate with age-related loss of bone mass density and increased bone resorption. Moreover, autophagy, a cellular behavior where cells enfold intracytoplasmic substrates by making a double-layer membrane around these substrates and secreting lysosomes for their degradation, maintains the activities and performance of cells through material recycling. However, with age, the autophagy mechanism slows down, resulting in BMD loss among the elderly population.

## 3. Relationship with Lifestyle

Bone mass is influenced by multiple factors, such as nutritional activity, physical activity, smoking, and alcohol intake. Diet is a key modifiable risk factor and contributes a significant part in achievement and maintenance of peak bone mass in early life. A good caloric intake, having an adequate amount of dietary protein, calcium, vitamin D, fruits, and vegetables, has a constructive influence, whereas a high caloric diet and heavy alcohol intake have been related to low BMD and significant incidence of fragility fractures. Dairy products, soy, fish, etc., are important dietary sources of calcium, fish and mushroom for vitamin D, and meat, fish, eggs, and beans for proteins. Evidence for other minerals and vitamins is not strong, but recent publications support the role of vitamins C and K for having a positive influence [20,21]. Natto, fruits, and vegetables are important sources of vitamins K and C [22,23]. Table 2 presents dietary sources of key nutrients and their roles in preventing OP, highlighting essential nutrients such as calcium, vitamin D, and protein, along with their primary dietary sources and the associated risks of deficiencies that contribute to decreased bone mineral density and increased fracture susceptibility. Physical activity is another modifiable risk factor that helps balance bone mass, muscle mass, and body balance. Adequate physical activity during early growing years helps achieve a higher peak bone mass and prevents OP while excessive physical activity may cause loss of bone mass, as seen among young female athletes by decreasing estrogen levels through decreased pulsatile secretion of gonadotropin-releasing hormone (GnRH) [22].

**Figure 2:**
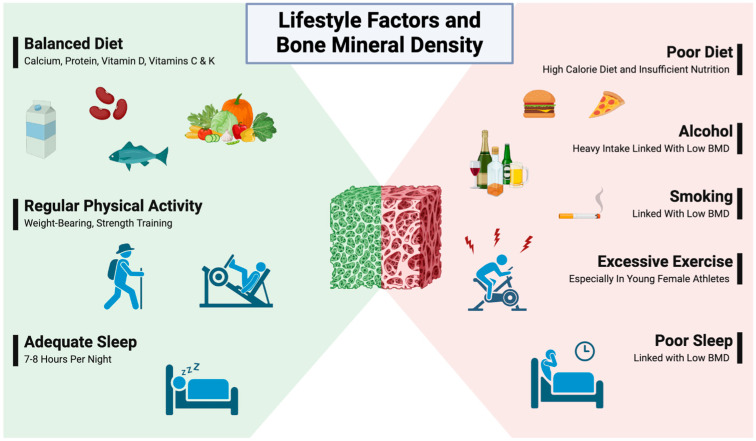
**Lifestyle factors implicated in bone mineral density changes.** A graphical illustration highlights some lifestyle factors implicated in bone mineral density (BMD) changes. Increased BMD is associated with regular physical activity, adequate sleep, and a balanced diet, including adequate protein, calcium, and vitamin D. Decreased BMD has been correlated with high-caloric diets, heavy alcohol use, smoking, excessive physical activity, and poor sleep quality. (Created in BioRender: BioRender.com/j42t268).

Recent studies have also supported the relationship between sleep and OP. Poor sleep quality has been suggested to induce OP by decreasing the number and activity of osteoblasts which results in decreased BMD. Increased sympathetic nervous system activity with resultant decreased bone formation and increased bone resorption is another suggested mechanism by which poor quality of sleep has been proposed to cause the progression of OP [24]. Figure 2 illustrates these lifestyle factors implicated in bone mineral density.

## 4. Renal Disease

### Chronic Kidney Disease and Renal Tubular Acidosis as Causes of Secondary Osteoporosis

**Figure 3:**
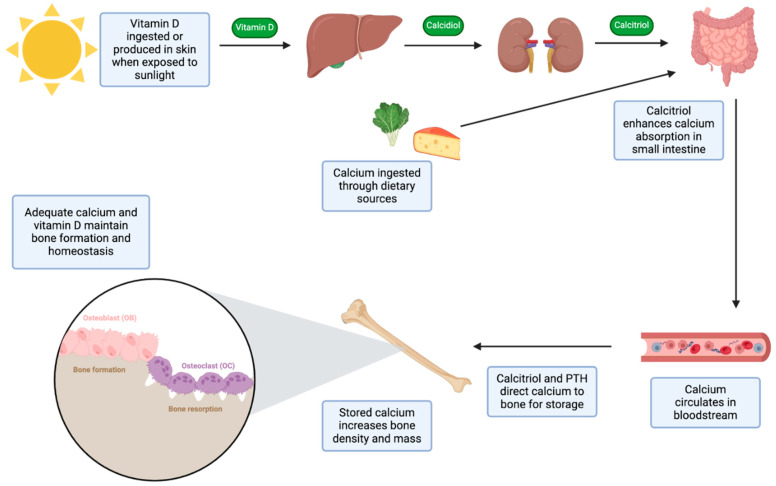
**Role of calcium and vitamin D in bone mass regulation.** A simplified schematic illustration highlighting key steps in calcium and vitamin D metabolism and function in regulating bone mass. The diagram illustrates the sequential steps from ingestion of calcium and vitamin D to the enhancement of bone mass. Starting with dietary calcium and vitamin D intake, vitamin D is activated in the liver and kidneys, converting it to calcitriol, the active form necessary for calcium regulation. Active vitamin D enhances calcium absorption in the intestines, increasing calcium levels in the bloodstream. The absorbed calcium is then directed to bones, where it is stored and incorporated into the bone matrix, promoting mineralization. This process increases bone density and strength, supporting overall skeletal health. (Created in BioRender: BioRender.com/z40i724).

Chronic kidney disease (CKD) is associated with the emergence of mineral bone disorder (MBD), leading to OP and increased susceptibility to fractures. Disruptions in mineral and humoral metabolism, coupled with alterations in bone structure, manifest early in the progression of CKD. CKD–MBD encompasses anomalies in calcium, phosphorus, parathyroid hormone (PTH), and/or vitamin D levels, as well as abnormalities in bone turnover, mineralization, volume, and linear growth or strength. The underlying pathophysiology of CKD–MBD is intricately tied to klotho expression, a membrane protein predominantly found in proximal and distal renal tubules. In the initial stages of CKD, the diminishing expression of klotho can elevate fibroblast growth factor-23 (FGF-23) levels, contributing to increased urinary phosphate excretion by reducing renal phosphate reabsorption. Phosphate retention further fosters the development of secondary hyperparathyroidism (SHPT) and hinders calcitriol production by impeding the expression of 1-alpha-hydroxylase in proximal tubules. Reduced calcitriol could then cause a concomitant decrease in calcium levels. Increased PTH can lead to increased bone breakdown, thereby affecting bone remodeling. Additionally, sclerostin and dickkopf1 are secreted by the kidney. As CKD progresses, sclerostin serum levels increase, which causes decreased bone formation by hindering wnt-induced signals through binding to low-density lipoprotein receptor-related protein (LRP5/6) increasing osteoclastogenesis by inducing RANK-L production. Raised serum dickkopf1 can also hinder bone development by preventing wnt-induced signaling through the same receptor acted on by sclerostin. The management of CKD–MBD includes lifestyle modification, including modifying dietary calcium and vitamin D, exercise, smoking cessation, avoidance of alcohol, and increased weight-bearing exercise. Management may also involve correcting biochemical abnormalities associated with CKD–MBD by lowering phosphate levels, reducing calcium intake, and/or using calcimimetics such as cinacalcet, which increases the sensitivity of the calcium-sensing receptor (CaSR) and reduces PTH secretion. Additionally, vitamin D analogs may be used to target the vitamin D receptor (VDR), further helping to regulate PTH levels and calcium–phosphate balance. The role of calcium and vitamin D in bone mass regulation is illustrated in Figure 3, highlighting their metabolic pathways from intake to bone mineralization, which is particularly relevant in managing CKD–MBD. Treatment involves parathyroidectomy, which has been shown in a previous study with a national cohort of long-term CKD patients to reduce bone turnover and improve BMD and decrease risk of fractures. Lastly, pharmacologic agents containing antiresorptive or anabolic properties may help retain BMD in CKD patients. Some antiresorptive agents have been studied in patients with CKD; however, they should be used with caution due to contraindication in patients with estimated glomerular filtration rate (eGFR) <35 mL/min. These agents include traditional bisphosphonates (BPs) such as zoledronic acid, risedronate, ibandronate, and alendronate. Other non-bisphosphonate antiresorptive agents used are denosumab, a monoclonal antibody to RANK-L, and raloxifene, a selective estrogen receptor modulator (SERM), which increases estrogenic activity in the bone. Anabolic agents are available for OP treatment but have not been studied in the context of CKD patients. Such agents include romosozumab, a monoclonal antibody against sclerostin, abaloparatide, and teriparatide [25]. Renal tubular acidosis (RTA) is a non-anion gap metabolic acidosis. Type 1 and type 2 RTA are the most common and are characterized by faulty secretion of hydrogen ions and compromised absorption of bicarbonate, respectively. Long-lasting uncorrected acidosis can contribute to MBD and subsequent OP. RTA involves the bone through acidosis-mediated inflated osteoclastic bone resorption. Additional factors that contribute to bone resorption dysregulation in proximal RTA include irregular renal control of phosphate, contributing to hypophosphatemia, and impaired vitamin D metabolism and function. Contributing factors of distal RTA include hypercalciuria and SHPT [26].

CKD-associated mineral and bone disorders (CKD–MBDs) introduce a unique challenge in secondary OP management due to complex alterations in calcium–phosphate metabolism and secondary hyperparathyroidism. However, most studies focus on advanced CKD stages, leaving early-stage CKD largely unexamined in the context of bone health. This gap may overlook opportunities for early intervention to prevent the progression of bone loss in CKD patients.

## 5. Drug-Induced Osteoporosis

### Glucocorticoid-Induced Osteoporosis

**Figure 4:**
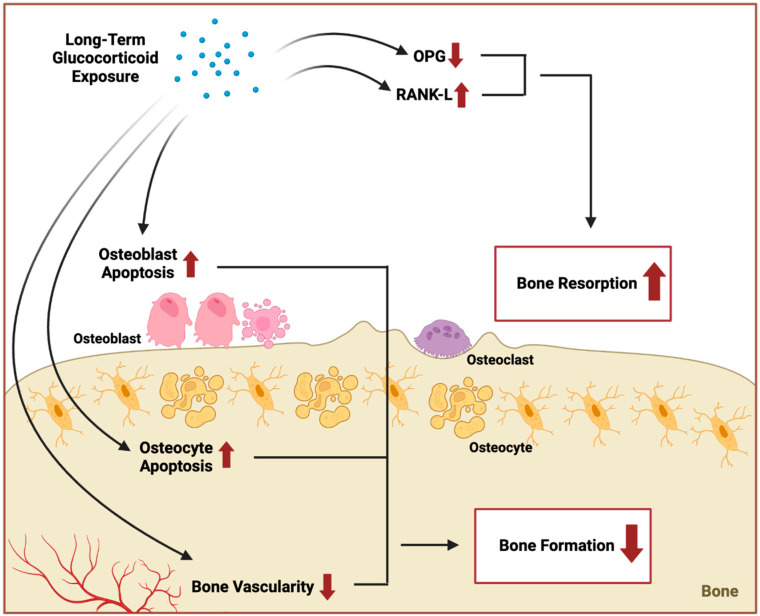
**Mechanisms of glucocorticoid-induced osteoporosis.** A simplified graphical illustration of the mechanisms of glucocorticoid-induced OP. Long-term glucocorticoid exposure reduces bone strength and formation by reducing bone vascularity and by increasing osteoblast and osteocyte apoptosis. Long-term glucocorticoid exposure increases bone resorption by increasing expression of RANK-L and decreasing OPG activity, which leads to increased osteoclast activation. (Created in BioRender: BioRender.com/c41q691).

Glucocorticoids (GCs) are the most common cause of medication-induced OP with an estimated 30% of patients treated with GCs suffering an osteoporotic fracture. The mechanism by which GCs induce OP is through the reduction in osteoblast (OB) and osteocyte (OC) lifespan/activity and reduction in the vascularity of bone, which may help explain the reduction in bone strength and mass (Figure 4). Glucocorticoids are anti-inflammatory medications used in the management of inflammatory non-infectious diseases including rheumatoid arthritis, systemic lupus erythematosus, organ transplantation, asthma, and malignancies [27]. On a molecular level, GCs increase expression of RANKL and decrease OPG levels (a RANKL decoy receptor) which increase OC differentiation and activation in the short term [28]. With long term GC use bone formation is reduced via increased OB and osteocyte apoptosis. This decrease in OBs may be mediated by increased levels of wnt/beta-catenin antagonist (dickkopf1 and sclerostin) via GCs. On a humoral level GCs can cause hypogonadotropic hypogonadism and reduction in intestinal calcium absorption and hypercalciuria, thus promulgating OP pathogenesis [28]. Non-pharmacological treatments include sufficient consumption of calcium and vitamin D, as well as weight-bearing exercise [27]. Bisphosphonates may also be used for the avoidance and management of glucocorticoid-induced OP (GIOP) through reducing OC lifespan and activity. Several publications have compared the effectiveness of BPs in the treatment of GIOP with initial randomized control trials (RCTs) showing significantly elevated BMD of the lumbar spine and hip compared to placebo treatment in both prevention and treatment studies [29]. Other studies further compared specific BPs such as zoledronic acid and risedronate with no alterations in incident fractures [27,30]. Previously only four medications were permitted for treatment of GIOP: alendronate, risedronate, zoledronate, and teriparatide. In May 2018, denosumab was accepted for the treatment of GIOP in men and women at elevated risk for fracture [28]. In a systematic review carried out by Yanbeiy and Hansen, denosumab was shown to increase lumbar spine BMD (2.32%, 95% CI 1.73%, 2.91%, *p* < 0.0001) and hip BMD (1.52%, 95% CI 1.1%, 1.94%, *p* < 0.0001) compared to BPs across three studies [28]. In another study by Wang and Li, a meta-analysis was conducted in an eastern Asian population in order to relate the efficacy of BPs, vitamin D, and a combination treatment for inhibiting and treating GIOP. Nine RCTs were included with an N of 545. They found that in contrast to vitamin D alone, BPs and vitamin D treatment significantly improved OP of the spine and femoral neck in their study population [31]. GCs also modify the intestinal microbiota composition. Gut microbiotas are recognized to aid in the regulation of bone density [31]. This association was tested in a study by Schepper et al. in C57/BI6J mice who were treated for eight weeks with GCs in the absence or presence of broad-spectrum antibiotic treatment. Long-term antibiotics (ABXs) prohibited GC-induced trabecular bone loss displaying the necessity of gut microbiota for GIOP. The dealing of GC-treated mice with a probiotic (*Lactobacillus reuteri*) prohibited trabecular bone loss. Furthermore, fecal transplant of GC-treated mice fecal material into recipient naive untreated wild-type mice caused bone loss. The authors of this study also noted GC contributes to intestinal barrier breaks [32]. While glucocorticoid-induced OP has well-documented pathophysiological mechanisms, discrepancies exist in the efficacy of different bisphosphonates across populations. The lack of large-scale, long-term trials on certain secondary OP forms, such as T2DM-induced OP, highlights the need for further research on treatment efficacy and safety.

## 6. Endocrinopathies

### 6.1. Diabetes-Induced Osteoporosis 

Type 2 diabetes mellitus (T2DM) is one of the most common metabolic disorders resulting from inadequate insulin secretion by pancreatic beta cells or insufficient responsiveness in insulin-sensitive tissues. Epidemiological studies have shown an increased frequency of osteoporotic fractures among patients with T2DM compared with healthy populations, underscoring a significant relationship between T2DM and osteoporosis. Chronic hyperglycemia in T2DM increases reactive oxygen species (ROS), which disrupts bone remodeling by impairing osteoblast function and enhancing osteoclast activity, leading to an imbalance in bone turnover and increased bone fragility. Additionally, iron overload is implicated in T2DM-related bone loss and is closely linked to ferroptosis—a regulated cell death pathway dependent on iron and ROS. In T2DM, iron overload may result from overexpression of divalent metal transporter 1 (DMT1) in osteoblasts, contributing to oxidative stress and exacerbating bone deterioration. Melatonin also affects bone metabolism and has been shown to serve as an effective endogenous antioxidant that indirectly stimulates antioxidant enzymes. Melatonin has been shown in rat studies to prevent OP and has also been reported to inhibit bone loss in perimenopausal women [33]. In a study by Ma et al., they reported that raised glucose induces ferroptosis via increased ROS, lipid peroxidation, and glutathione exhaustion in T2DM OP [34]. They also presented that melatonin meaningfully reduced the level of ferroptosis and enhanced the osteogenic capacity of MC3T3-E1 cell lines by stimulating the nuclear factor erythroid 2-related factor 2/heme oxygenase 1 (Nrf2/HO-1) pathway in vivo and in vitro [34]. Those with T2DM may be theoretically treated with metformin. Metformin has also been shown to play a role in the promotion of OB differentiation through phosphorylation of extracellular signaling molecules and by reducing hyperglycemia which decreases intracellular ROS and advanced glycation end products in collagen [35]. Therefore, melatonin and metformin may have protective roles against osteoporosis in T2DM patients by targeting key mechanisms associated with bone degradation.

While T2DM is strongly linked to increased fracture risk, understanding the precise mechanisms of bone loss remains challenging due to variability in T2DM pathophysiology, patient age, and duration of disease. The role of advanced glycation end products (AGEs) in collagen deterioration is well-documented, yet the extent to which AGE accumulation versus hyperglycemia directly impacts bone quality is still debated. Additionally, the metabolic control of diabetes (e.g., insulin versus non-insulin treatments) may differentially affect bone health, although studies directly comparing these effects are sparse.

### 6.2. Hyperthyroidism

Thyroid hormones are mandatory for skeletal growth and attainment of peak bone mass. T3 regulates bone turnover and BMD; therefore, a balance of thyroid hormone levels is necessary to sustain healthy bone. Population studies show that both hypothyroidism and hyperthyroidism are linked with an amplified risk of fracture [36]. Unrecognized severe hyperthyroidism influences bone mass and raises the likelihood of high bone turnover, contributing to the development of OP, particularly in post-menopausal women. Subclinical hyperthyroidism is defined as low thyroid stimulating hormone (TSH) and free hormones within the reference range and can oftentimes go undetected when asymptomatic [37]. A study by Baliram et al. revealed that not only does a clinically low thyroid hormone level contribute to OP but also decreased TSH signaling and thus therapeutic suppression of TSH to very low levels may contribute to bone loss in some people [38]. There are different etiologies that may lead to hyperthyroidism, such as treatment with antiviral drugs, Grave’s disease, pituitary adenoma, thyroiditis, radiation, and intake of excessive thyroid hormone or iodine [39]. Treatment for hyperthyroidism would involve removing the etiologic pathology, such as antithyroid drugs, radioactive iodine therapy and surgery in the case of Grave’s disease, and pituitary adenoma, or removal of the causative agent in the case of excesses of thyroid hormone or iodine, radiation, or antiviral drugs.

### 6.3. Hypogonadism/Hypopituitarism

One of the foremost secondary causes of OP in men is hypogonadism, which is reported in up to 20% of men with symptomatic vertebral fractures and 50% of elderly men with hip fracture [40]. Androgens in men contribute a significant part in the conservation of healthy bone mass via maintenance of cancellous bone and stimulation of periosteal bone apposition. Aromatization of testosterone to estradiol plays a key role in maintenance of bone homeostasis in males. Drugs accepted for the management of OP in men include anti-resorptive bisphosphonates: alendronate, risedronate and zoledronic acid, the antiresorptive drug denosumab, and the bone-forming agent teriparatide. Although data are limited in men in terms of these drugs, the existing data suggest that treatment effects in men are comparable to those witnessed in studies with postmenopausal women. There may be cases where hypogonadism is exacerbated by other treatments or conditions. For instance, BPs may be indicated in the context of androgen deprivation therapy for the treatment of non-metastatic prostate cancer as well as GIOP in men. Lastly, testosterone treatment may be required in men with clinically significant hypogonadism, but the presence of OP is not sufficient to warrant hormone replacement therapy for testosterone [41].

### 6.4. Eating Disorders

Eating disorders are psychiatric disorders which have been associated with a higher possibility for low BMD and subsequent fractures. The etiologic factor of low BMD in eating disorders stems from malnutrition, fluctuations in body composition, and hormonal changes. Two of the most studied eating disorders that play a part in the development of secondary OP are anorexia nervosa (AN) and bulimia nervosa (BN). These two psychiatric disorders are most prevalent in adolescent-aged females, where undernutrition can interrupt achievement of peak bone mass, causing long-term skeletal problems. Lower fat mass (particularly subcutaneous fat) may constitute a part in the pathogenesis of OP in patients with eating disorders. Subcutaneous fat serves as a site of leptin production, a hormone with anabolic effects. Studies have also shown that marrow energetics and the hormonal environment influence stromal progenitor stem cells in the marrow to differentiate into either OBs or adipocytes. It has been shown that marrow adipose tissue (MAT) is higher in women with AN compared to controls, associated with lower BMD measures. Multiple endocrine axes may be disrupted in those with eating disorders. In women, a decrease in estrogen due to disruption of the hypothalamic–pituitary–gonadal axis will increase OC bone resorption, along with decreased bone formation by causing the disinhibition of sclerostin and preadipocyte factor-1 (a negative regulator of osteoblastogenesis in the marrow). First-line treatment for those with secondary OP and an eating disorder is weight gain and resumption of menses. Nutritional supplementation may aid in restoring some vitamins and minerals missing from the diet in patients with eating disorders. Such supplements should include activated vitamin D, calcium, and vitamin K supplementation for bone health. Hormone replacement therapy may be instituted with the pretext of pituitary–gonadal axis dysfunction. In this case, estrogen replacement would be warranted since normal estrogen status is essential for pubertal bone accrual and to prevent bone loss in adult women with AN. Replacement of insulin-like growth factor (IGF-1) with recombinant IGF-1 may be administered since decreased IGF-1 levels correlate with reductions in surrogate markers of bone formation and lower BMD. Lastly, BPs can be used in these patients as they are the most prescribed drugs to treat OP, and studies have shown that they increase bone density at the spine and hip in adult women with AN over 12 months (risedronate) [42].

### 6.5. GH Deficiency

Adults with growth hormone deficiency (GHD) have impairments of the bone related to variations in bone mass due to reduced bone mineralization, which increases fracture risk [43]. Several systematic elements have a negative effect on GH secretion, including IGF-1 and GCs, which could exacerbate low GH levels in those taking steroids. The effects of GH were elucidated in a seminal study by Harris and Heaney that revealed exogenous GH increases skeletal mass in dogs. IGF-1 of hepatic origin produces the molecular mechanisms underlying this phenomenon in response to GH. Numerous hormones, including parathyroid, cortisol, and estrogen, also impact IGF-1 synthesis in bone osteoblasts, which is then secreted in a paracrine and autocrine fashion [44]. There has been a sexually dimorphic effect of GHD on BMD with affected men suffering a lower BMD than women; this may be due to the anabolic effects of estrogen on bone mass. Although GH replacement has been demonstrated to show a linear influence on the growth of children with GHD, many studies have also shown GH replacement in adults with GHD for 18 months or longer has encouraging effects on BMD, which was further established in a 10-year prospective study showing a constant rise in BMD. However, more long-term clinical trials are required to confirm this phenomenon [44]. Other studies have also exemplified that long-term GH replacement at physiological doses increased BMD in men with adult-onset GHD, but the data were inconclusive on the therapeutic benefits conferred to bone effects in women with GHD [43].

### 6.6. Acromegaly 

Acromegaly is described by surplus growth hormone with resultant elevated IGF-1 levels commonly from pituitary adenoma origin. Untreated acromegaly causes increased mortality and morbidity, including increased incidence of hypogonadism and diabetes mellitus, which could both further exacerbate BMD [45]. Interestingly, a study by Mazziotti et al. reported that acromegaly patients with diabetes had an elevated prevalence of vertebral fractures (72%) than those without diabetes (43.6%) [46]. Those with acromegaly are an at-risk population for vertebral fractures with a prevalence of radiographic vertebral fracture of 39–59% in cross-sectional studies of these patients. The mechanisms leading to OP in the background of acromegaly consist of higher GH and IGF-1 levels. It has been suggested via in vitro studies that IGF-1 promotes RANK-L production and osteoclastogenesis, disrupting the delicate balance of resorption and anti-resorptive mechanisms in bone homeostasis. Alternatively, GH promotes osteoprotegerin upregulation, which is a decoy receptor for RANK-L. Despite the increased fracture risk displayed in some studies, other studies demonstrate discordant results on the effects of acromegaly on BMD. Some studies suggest an increase in BMD, and others a decreased BMD. In a study of 36 women with acromegaly, individuals with active disease had a higher BMD in contrast to those with controlled disease. Ironically, vertebral fracture risk was greater in patients with active disease, although they had a higher BMD, suggesting other mechanisms at play causing bone fracture. In contrast, one prospective study exemplified that those with acromegaly had a lower BMD than non-acromegalic controls [45]. The first-line treatment for acromegaly is trans-sphenoidal surgery which removes the compressive tumor source which directly addresses the increased GH production/secretion. When surgery fails, medical treatment includes somatostatin analogs in an attempt to bring GH closer to homeostatic levels and radiotherapy to ablate the offending tumor. Lastly, in patients resistant to somatostatin analogs there is a novel GH antagonist called pegvisomant that can be used [47].

## 7. Gastrointestinal Disease

### 7.1. Celiac Disease 

Celiac disease (CD) is an autoimmune disease of the small bowel due to gluten sensitivity. This inappropriate response to gluten promotes an exaggerated inflammatory response and subsequent mucosal damage [48]. Celiac disease may aggravate the progression of bone loss and screening should be implemented in this patient population as it may be the only sign of undiagnosed CD. The pathogenesis of CD includes raised interleukin (IL)-1beta in patients with a concomitant genetic predisposition to OP. Of the IL-1 system of IL-alpha, IL-1beta, and IL-1 the former two cytokines are powerful promoters of bone resorption by prompting proliferation and differentiation of OC precursors and mature active OCs. Genetic predispositions play a role in the severity and threshold of initiation of OP. Studies have shown that different allelic variants in the IL-1 system have associations with decreased BMD in postmenopausal women. One study, which included 220 postmenopausal Korean women between the ages of 48 and 70, reported that of those carrying the A2 allele of the IL-1 receptor agonist gene, BMD was inferior to those that did not carry the allele. Another genetic polymorphism affecting bone remodeling in CD is the castro-interacting zinc finger protein (Ciz) gene. This protein has been shown to inhibit bone formation without affecting resorption and has been identified as a potential therapeutic target whose inhibition may increase BMD [49,50]. Other effects of CD on bone metabolism also stem from improper absorption of calcium and vitamin D [51]. Management of CD is through a strict gluten-free diet to remove the inflammatory excitatory molecule from coming into contact with intestinal mucosa. This is the only recommended treatment for those with CD currently, as corticosteroids only benefit a small percentage of patients with CD and may worsen subsequent osteoporotic conditions [48]. 

### 7.2. Inflammatory Bowel Disease

Inflammatory bowel disease (IBD) is a chronic inflammatory disease of the gastrointestinal (GI) tract which includes Crohn’s disease and ulcerative colitis. These disorders are due to an exaggerated inflammatory response to certain foods and intestinal flora and may be associated with a genetic predisposition to such diseases [52]. IBD can lead to damage of the organization and function of the GI tract that can lead to the development of OP, especially in the elderly population. The estimated prevalence of OP in IBD has been reported to be 42%, with slighter decreases in BMD being described in 70% of those with IBD. The pathophysiologic mechanisms underlying the bone loss in IBD may be specific to the underlying inflammation associated with the disease as well as magnitudes of the disease process itself, including variations in diet, reduction in muscle mass, decreased absorption of nutrients, vitamins, and minerals, reduced weight-bearing exercise, and usage of osteotoxic medications. More specifically, inflammation in IBD is mediated mainly through the over-activation of T cells, which increases cytokines known to stimulate the production and maturation of OCs. Management of OP in those with IBD includes modifiable lifestyle factors such as avoiding excessive alcohol and drugs, smoking cessation, and doing weight-bearing exercises. Limiting the use of steroids can also be helpful in preventing bone loss in this population. Those with IBD at risk for OP may also benefit from increased supplementation with vitamin D and calcium. These supplements have been shown in previous studies to increase BMD in those with low baseline BMD and IBD, although it did not reduce the danger of decreased BMD in those with IBD using GCs. Bisphosphonates may be prescribed in the treatment of IBD-associated OP with risedronate reported to considerably improve BMD in post-menopausal women with IBD and thus decrease the frequency of vertebral fracture by two thirds in this population [53]. 

### 7.3. Hemochromatosis 

Hemochromatosis (HHC) is a condition that promotes excess iron deposition through excessive intestinal iron absorption and, thus, multiple organ dysfunction. These organs can vary widely and include the liver, joints, pituitary glands, gonads, and heart. Therefore, there may be multiple etiologies contributing to secondary OP in these patients related to hypogonadism, pituitary dysfunction, and liver dysregulation [52]. Raised hepatic iron concentrations are linked with low femoral BMD among male patients with HHC. It is important to note that iron overload itself is associated with OP, demonstrated by its improvement with iron removal via phlebotomy. The pathologic mechanism underlying the disease that leads to OP can be attributed to iron infusions elevating FGF-23 secretion of osteocytes with resultant renal phosphate wasting, calcitriol deficiency, and secondary hyperparathyroidism all shown to affect bone remodeling and homeostasis. Excess iron has also been shown to inhibit both hydroxyapatite crystal growth and OB cell proliferation, differentiation, and mineralization in vitro, possibly leading to lower BMD [54]. The primary therapy for HHC is phlebotomy, usually performed once or twice a week. Once iron levels normalize, lifelong, but a lesser amount of, repeated phlebotomy (usually 3–4 times a year) is required. It is also suggested in those with HHC that alcohol drinking be strictly avoided because it can speed up liver and pancreatic toxicity. Chelation therapy has not been shown to be effective in HHC, and erythropoietin combined with phlebotomy has been at times administered to sustain hemoglobin concentration while promoting iron mobilization. Patients with end-stage liver disease may need liver transplantation; however, previous studies have shown that when compared to non-HHC patients, those with HHC who undergo liver transplantation have lower survival rates [55].

### 7.4. Chronic Liver Disease

Liver disease, particularly liver cirrhosis and cholestatic liver diseases, predispose one secondarily to OP. In addition, OP is also observed in chronic liver diseases such as chronic viral hepatitis, nonalcoholic fatty liver disease, and alcoholic liver disease. The mechanisms underlying OP in patients with chronic liver disease are complex and not completely understood. A deleterious inflammatory state associated with chronic liver disease may be implicated in the pathogenesis of OP in these patients. For example, in viral hepatitis an activated immune response and release of resorption activating cytokines can precipitate OP. Direct or indirect toxic effects on OB differentiation and survival may also be observed in some liver diseases. Diseases that produce these effects are primary biliary cholangitis (PBC) and primary sclerosing cholangitis. In these diseases raised bilirubin levels exert a negative effect on OBs. Additionally, sclerostin from osteocytes hinders wnt/beta-catenin signaling during initial bone disease in patients with cholestatic diseases, which prevents wnt from binding to LPR5/6 receptors preventing OB differentiation. A decrease in trophic factors as a result of liver disease may also contribute to the pathogenesis of OP. IGF-1, an anabolic hormone produced mainly by the liver and in response to GH stimulation, is crucial in bone growth, reduces OB apoptosis, and promotes osteoblastogenesis [56]. Liver transplantation also represents the dynamic interplay between liver function and bone homeostasis. Liver transplant recipients experience accelerated bone loss during the period following transplantation. Post-operative fractures are observed in up to 35% of patients after liver transplant associated with high bone turnover. In addition, this accelerated loss may be due to side effects of immunosuppressive therapy associated with liver transplant, which would be influenced by doses of GCs combined with cyclosporine or tacrolimus, other drugs that have a negative impact on bone formation [56]. Treatment of OP in patients suffering from liver disease includes calcium and vitamin D supplementation in addition to pharmacological therapies limited to those without significant negative impact on liver function. Increases in bone formation have been shown in patients taking intermittent parathyroid hormone as well as sodium fluoride and etidronate (a bisphosphonate) where a subtle increase in vertebral BMD was found in PBC patients. However, sodium fluoride and etidronate are no longer recommended for secondary OP management and future studies are required to demonstrate the safety and efficacy of sodium fluoride in patients with cirrhosis. Given that bone changes are present in patients with chronic liver disease (CLD), further investigation is essential to rule out other direct etiopathogenetic mechanisms involved [57]. Additional therapies include inhibition of OC activation through anti-resorptive agents such as selective estrogen receptor modulators (SERMs) and RANK-L inhibitors. Lastly, gut dysbiosis has been displayed in patients with chronic liver disease when compared to healthy individuals, which has been shown to influence the development and function of the host immune system. Therefore, probiotic administration can help reduce the manifestation of several pro-inflammatory and osteolytic cytokines, such as TNF-alpha and IL-1beta [58].

## 8. Hematologic Disorders

### Monoclonal Gammopathy of Uncertain Significance

Monoclonal gammopathy of undetermined significance (MGUS) is characterized by the presence of monoclonal paraprotein in the bloodstream, without the distinct end-organ damage seen in multiple myeloma [59]. The prevalence of MGUS rises with age, reaching 8.9% in males and 7.0% in females over 85 years. The predominant immunoglobulin isotype is IgG (observed in 68.9% of cases), with kappa as the most common light chain type (62.0%). The annual progression rate to myeloma among MGUS patients is 1%. Uncertainty surrounds the impact of MGUS on bone mineral density, bone turnover, and fracture incidence. The current comprehension of bone lesion pathogenesis in myeloma is linked to excessive bone resorption. Myeloma cells induce RANKL expression in stromal cells through direct cell-to-cell contact, involving adhesion systems such as very late antigent-4 (VLA4) integrin on myeloma cells and vascular cell adhesion molecule-1 (VCAM1) on stromal cells. Through interaction with CD44, myeloma cells can prompt endothelial cells to express RANKL. Subsequently, RANKL interacts with RANK receptors on the surface of osteoclast (OC) progenitors, leading to OC differentiation. Additionally, myeloma cells decrease OPG gene transcription in OCs and stromal cells. The release of syndecan (CD138) by myeloma cells combines with OPG, facilitating its internalization and lysosomal destruction. Macrophage inflammatory protein-1 (MIP-1)alpha/beta, chemokines from the same family, stimulate monocytes, resulting in increased OC activity in myeloma patients. OC expresses CCR5 which binds to MIP-1alpha, which in turn activates the final steps of OC progenitor differentiation, and both chemokines promote RANKL expression by stromal cells further increasing OC activity. In a study by Abrahamsen et al. (2005) consisting of 799 patients with suspected OP, 4.9% of patients with established OP had a monoclonal peak contrasting to only 2.2% of patients without OP. Similar studies show patients with hip fractures had a 6% prevalence of MGUS combined; studies suggest that MGUS may lead to bone loss and OP fractures [60,61]. Studied treatments in this relatively small population of patients have been sparse. One RCT study performed on 163 patients with asymptomatic myeloma, in which 81 patients received zoledronic acid and 82 patients received a placebo, found that there was no significant difference in rate of myeloma progression, monoclonal component level, or proportion of plasma cells in the bone marrow [62]. 

## 9. Systemic Mastocytosis

Systemic mastocytosis (SM) is an aggressive disorder caused by the release of numerous vasoactive cell mediators due to excessive activity of mast cells, introducing a wide variety of symptoms [63]. SM affects the skeletal system and puts patients in danger of OP and fractures. Patients suffering from SM have an overall 20% to 28% prevalence of OP compared with 8.3% in the general population. The mechanism of pathogenesis is not clearly defined in terms of the association between SM and OP. However, there is aberrant bone remodeling. This aberrant bone remodeling is purported to be associated with exacerbation of OC activity, an imbalance in signaling by OBs, and increased cytokine production by mast cells such as IL-6, IL-1, TNF-a, histamine, and tryptase resulting in increased bone resorption. One study including 50 patients with SM within a North American cohort found that 74% had a fracture history and 56% had a history of minor trauma-induced or spontaneous fractures, which was slightly higher than the comparison European cohort [64]. Another study conducted retrospectively in 8392 patients with OP found that of the 1374 patients from the cohort undergoing diagnostic bone biopsy, 43 patients had SM. This cohort represents 0.5% of those diagnosed with OP who also had SM; however, patients with SM suffered considerably more vertebral fractures in contrast to other patients in the bone biopsy cohort that did not have SM (4.4 ± 3.6 versus 2.4 ± 2.5 vertebral fractures, *p* < 0.001) [65]. Treatment of the primary disease is paramount in SM. The approach to SM depends on symptoms and subtypes of the disease, with treatment including antihistamines, antileukotriene drugs, and omalizumab (anti-IgE). For concurrent OP and fractures, regular consumption of calcium and vitamin D supplements are recommended in combination with pamidronate and low-dose interferon alfa [63]. 

## 10. Beta Thalassemia Major

Beta thalassemia major (BTM) is caused by mutation of the beta-globin gene, resulting in the absence of beta chains. BTM presents clinically as jaundice, growth retardation, hepatosplenomegaly, endocrine abnormalities, and severe anemia requiring lifelong blood transfusions [66]. BTM-associated OP is a complex condition that involves distinct acquired and genetic influences that produce its pathology. Ultimately, BTM contributes to imbalances in bone remodeling by inhibiting OB activity and promoting OC activity. OP fractures in thalassemic patients are exceedingly common (up to 44%). Other pertinent genetic factors associated with BTM are polymorphisms at the Sp1 site of the collage type Ia1 (COLIA1) gene and vitamin D receptor. Additionally, acquired factors such as primary disease, which activates bone marrow expansion and iron overload, contribute to complications of the disease and lower BMD. Furthermore, secondary factors produced from the primary factors likely include hypogonadism, GH and IGF-1 deficiency, diabetes, vitamin D deficiency, and kidney dysfunction which all contribute to dysfunction in bone remodeling [67]. Treatments depend on the degree of thalassemia and include frequent blood transfusions, chelation therapy due to chronic transfusions, stem cell transplant, gene therapy, splenectomy, as well as diet and exercise [68]. Also, OP management in patients with BTM is mainly confined to BPs with limited clinical data on its treatment with teriparatide, denosumab, and strontium ranelate [67]. Other studies have suggested alternatives not well studied or defined in the literature, such as silymarin derived from *Silybum marianum* L. (milk thistle), shown to increase bone formation by increasing the proliferation of OBs and inhibition of OC formation via attenuating signaling cascades associated with RANK-L and TNF-alpha [69].

## 11. Autoimmune Disorders

### 11.1. Rheumatoid Arthritis 

Rheumatoid arthritis (RA) is an autoimmune disorder of the joints characterized by inflammatory arthritis as well as extra-articular involvement [70]. RA causes an increased risk for OP fractures with associated BMD loss. Nearly one third of the RA population is affected by OP, which makes it a major risk factor for skeletal fragility status which is precipitated by the disease itself, medications used to treat it (glucocorticoids), and changes in body composition that raise the risk of frailty and fractures. Although there have been attempts to utilize alternative treatments for RA other than GCs, which should indicate a decline in OP in the RA population, some studies, particularly meta-analyses, show that fracture risk in RA still remains high [71,72]. Anti-citrullinated protein antibodies (ACPAs) implicated in the pathogenesis of the local joint erosion and systemic bone loss accompanied by RA have been hypothesized to increase the activity of OCs. Studies confirm this by showing that ACPAs that are present years before RA onset are still associated with findings of low BMD. Inflammation in RA also contributes to the development of OP as inflammatory cytokines promote bone resorption by activating OCs and inhibiting OB activity. Helper T cells of the Th-17 subtype play a significant role in osteoclastogenesis in rheumatoid arthritis (RA) through the secretion of the pro-inflammatory cytokine IL-17. In the context of osteoimmunology, IL-17 upregulates the expression of RANKL on synovial fibroblasts and osteoblasts, which then stimulates osteoclast differentiation and activity, leading to increased bone resorption and joint damage [73]. Additionally, IL-17 directly induces osteoclast formation from monocyte precursors in the presence of pro-inflammatory factors, creating a direct pathway for bone erosion in RA without the need for osteoblast interaction [74]. This osteoimmunological mechanism contributes to OP secondary to RA, as sustained Th-17/IL-17 activity promotes chronic inflammation, enhancing osteoclastogenesis and accelerating bone loss associated with RA. Treatment of RA includes inhibition of specific inflammatory cytokines such as IL-6, as well as TNF-alpha, which promotes both improved BMD and physical functioning. Treatment of RA includes non-biologic disease-modifying antirheumatic drugs (DMARDs) which include hydroxychloroquine (HCQ), azathioprine (AZA), and cyclosporine, the last of which is also associated with exacerbation of OP. Rituximab, a non-TNF DMARD, is also used in RA to suppress CD20+ B cells and decrease the immune response; similarly, abatacept works to inhibit T cell activation and decrease the immune response [70]. In regards to OP in the setting of RA, bisphosphonates such as zoledronate, denosumab, SERMs, and teriparatide can be used [75]. 

### 11.2. Systemic Lupus Erythematosus

Systemic lupus erythematosus (SLE) is a systemic autoimmune disease with multisystem involvement and is associated with significant morbidity and mortality. It involves loss of self-tolerance against self-antigens resulting in the production of pathologic autoantibodies that cause tissue injury systematically [76]. Over 90% of those with SLE are women, with inherent gender-specific risk for OP. Chronic inflammation in SLE contributes to bone loss by upregulating OC activity and inhibiting that of OBs. RANKL and TNF-alpha increase OC activity and maturation, and IL-1 and IL-6 also contribute a similar role in bone metabolism. Low-density lipoprotein can be raised in SLE and is considered to contribute in the disease phenomenon by activating T cells, thereby promoting RANKL and TNF as well as downregulating OB formation. Furthermore, hypovitaminosis D is common in SLE, and low vitamin D has been associated with low BMD and increased OP disease severity. This hypovitaminosis D may be due to the precautions the patients with SLE must take to avoid sunlight which produces ultraviolet radiation and can trigger a disease flare. Associated symptoms of SLE, such as lupus nephritis, can also contribute to the development of OP with chronic kidney disease increasing the risk of hyperparathyroidism resulting in disturbed calcium and vitamin D homeostasis [77]. Management of SLE focuses on preventing organ damage and achieving remission, as well as the selection of treatment targets for organ system involvement. However, treatment ranges from the least invasive treatment with NSAIDs and antimalarials to extensive treatment with cytotoxic drugs and corticosteroids that could further exacerbate OP and/or initiate it. OP-specific treatment in SLE includes vitamin D and calcium supplements, as well as BPs which are the mainstay of OP treatment [76]. One meta-analysis showed that BPs used in patients with rheumatic disease on GCs revealed reduced fracture rate and improved BMD in both prevention and treatment trials, which indicates that even with GC treatment, BPs may be efficacious. Additionally, teriparatide, denosumab, raloxifene, and calcitonin may be used as alternative therapies to bisphosphonates [77]. 

### 11.3. Ankylosing Spondylitis 

Ankylosing spondylitis (AS) is a chronic, inflammatory disease of the axial spine. It is characterized by progressive spinal stiffness and can produce impaired spinal mobility, postural abnormalities, and hip pain [78]. Bone complications include new bone formation in the form of syndesmophytes (a radiographic hallmark of AS), erosions, generalized OP, and vertebral fractures. Data suggest that OP is prevalent in 25% and vertebral fractures in 10% of the patients with AS. There may be delays in diagnosis of OP in AS as spinal BMD can be falsely raised due to ligamentous calcifications, sclerosis of the vertebral margins, and syndesmophyte formation. As with most inflammatory rheumatic conditions, the pathologic mechanism is underscored by inflammation associated with IL-6 and TNF-alpha that can amplify osteoclastogenesis through induction of RANKL and inhibit OBs via the inhibition of the wnt pathway, thereby producing a bone-resorptive environment. Additional molecular factors that are associated with AS are more complicated. These factors include Dickkopf-1 DKK-1 and sclerostin. DKK-1 is a soluble inhibitor of the wnt pathway and serum levels of this protein in AS patients are reduced, which causes wnt upregulation and subsequent OB differentiation. Serum sclerostin, an inhibitor of OBs, is also reduced in patients with AS. These two factors combined dysregulate the balance of bone remodeling in AS to an unregulated state of new bone formation. However, the process of new bone formation is site-specific in AS, occurring at vertebral edges (syndesmophytes), entheses, and joint margins. Therefore, it is the background inflammation that contributes towards OP, even with new bone forming at some sites. Treatment of OP in AS patients follows the same general guidelines as with the management of OP in any other patient [79]. There are no clinical trials evaluating the effectiveness of calcitonin, testosterone, vitamin D metabolites, or HRT in the setting of AS. However, there are data on the use of BPs for the treatment of OP specifically in AS. Pamidronate was initially used to reduce inflammation as this was a plausible mechanism for AS treatment-based animal models. Two open-label trials of pamidronate in patients with AS showed little change in inflammatory markers but did show improvement in bone turnover markers. Therefore, there may be some efficacy in using bisphosphonates for OP in the setting of AS [79]. Another treatment explored in the literature is anti-TNF agents that have complementary effects on bone and inflammation. One study, a phase III randomized placebo-controlled trial of infliximab on 279 patients with AS, showed a 2.5% increase in spinal BMD. However, this study fails to determine the effects of infliximab on fractures [80]. 

### 11.4. Multiple Sclerosis 

Multiple sclerosis (MS) is an autoimmune disease of the central nervous system (CNS) characterized by chronic inflammation, demyelination, gliosis, and neuronal loss [81]. MS is associated with decreased bone mass and elevated risk of developing OP. Treatment for MS via short-term intravenous glucocorticoids or subcutaneous/intramuscular injections of corticotropin may cause decreased bone formation, but the effect is usually reversible, and OP in MS patients may develop independent of using these agents. Pathogenesis of OP in MS may be associated with progressive immobilization/decreased activity, vitamin D deficiency, and skeletal muscle atrophy. Genetic susceptibility and environmental risk factors both contribute to MS, and warmer locations and cod liver oil supplements have been shown to be protective and reduce the risk of MS. Furthermore, immune cells such as macrophages, T cells, B cells, and dendritic cells express VDRs and can respond to active vitamin D. Studies have also shown that activated vitamin D may enhance the activity of suppressor T cells that preserve self-tolerance. In the context of genetic predispositions of MS, sequence analysis localizes a single MHC vitamin D response element (VDRE) to the promoter region of Human Leukocyte Antigen DRB1 (HLA-DRB1) which is conserved in MS HLA-DRB1 homozygotes; thus, expression of MS-associated MHC class II allele HLA-DRB1*1501 is regulated by vitamin D. Polymorphisms of the VDR may influence vitamin D function and metabolism [82]. One study that assessed the DNA of 77 MS patients and 95 healthy controls identified Bsm I and Apa I polymorphisms of the VDR gene showed that the AA genotype and the [A] allele were significantly more prevalent in MS patients than in controls, suggesting increased risk [83]. Lastly, vitamin D binding protein (DBP) is a carrier protein of vitamin D and has several other biologically important functions, including macrophage activation and chemotaxis. DBP is highly polymorphic, with three common alleles and over one hundred different variants. In a case-control study of two polymorphisms (codon 416 and codon 420) in the DBP gene utilizing two populations, Japanese patients with MS and healthy controls showed these polymorphisms have an association with the occurrence of MS. This suggests that DBP may be a potentially useful biomarker for the diagnosis and target of treatment for MS [84]. The management of MS includes disease-modifying therapeutics such as glatiramer acetate, dimethyl fumarate, fingolimod, interferon beta preparations, natalizumab, ocrelizumab, and mitoxantrone. Treatment of OP in MS should be primarily started with vitamin D supplementation as MS may predispose one to lower baseline levels of vitamin D than normal. Other treatments should follow OP guidelines with the use of bisphosphonates; however, there is not much literature looking into the efficacy of these treatments specifically for the MS population.

### 11.5. Genetics

Some non-modifiable causes include genetic diseases, which may also increase the risk of secondary OP. Thalassemia, an autosomal recessive disorder of red blood cells and their related bone disease, is characterized by bone deformity, reduced BMD, increased risk of fractures, and pain in bones. The thalassemia-related bone disease affects younger people, and in patients with transfusion-dependent thalassemia, the degree of bone disease is even more severe with 11.6% fractures. OP is also a complication associated with Ehlers–Danlos syndrome (EDS), which comprises a group of inherited connective tissue diseases, characterized by mutations in collagen-encoding genes [85].

## 12. Conclusions

Secondary causes of OP, which include endocrine disorders, renal disorder, gastrointestinal disorder, hematological disorder, autoimmune disorder, and the adverse effect of drugs, are highly prevalent and often curable. This article provides a framework for the approach and identification of these etiological classes that can help physicians, especially primary care physicians, in the early identification and prevention of bone loss. Most currently available pharmaceutical interventions are focused on excessive bone resorption or inflammatory cytokines [86]. Also, the current guidelines on the management of OP do not include secondary OP [85]. Hence, further research should be considered to understand molecular mechanisms involved in osteocyte senescence to develop an innovative intervention that targets the prevention of these mechanisms for improved outcomes [86]. Moreover, studies on the detailed function of osteomacs in the bone remodeling process would be helpful in developing new approaches for bone osteolytic diseases. Furthermore, approaches that focus on inflammation reduction can have a positive effect on bone health in aging populations [86].

## Figures and Tables

**Table 1:** Diagnostic categories for osteoporosis as per WHO.

**Lifestyle Factors:**
Nutritional deficiency (protein, calcium, vitamin D, fruits and vegetables, vitamins C and K)
Physical inactivity, excessive physical activity/female athletes
Poor sleep quality
**Renal disease**
CKD and renal tubular acidosis
**Drug inducements**
Corticosteroids
**Endocrinopathies**
Diabetes mellitus
Hyperthyroidism
Hypogonadism/hypopituitarism
Eating disorders
GH deficiency
Acromegaly
**Gastrointestinal diseases**
Celiac disease
Inflammatory bowel disease (ulcerative colitis and Crohn’s disease)
Hemochromatosis
Chronic liver disease
**Hematologic disorders**
Monoclonal gammopathy of uncertain significance
Systemic mastocytosis
Beta thalassemia major
**Autoimmune disorders**
Rheumatoid arthritis
Systemic lupus erythematosus
Ankylosing spondylitis
Multiple sclerosis
**Genetic diseases**
Thalassemia-related bone disease
Ehlers–Danlos syndrome

**Table 2:** Dietary sources of key nutrients and their role in preventing osteoporosis.

Nutrient	Dietary Sources	Role in Bone Health	Impact of Deficiency
**Calcium**	Dairy products (milk, cheese, yogurt), fortified cereals, leafy greens, soy, almonds, fish with bones (e.g., sardines)	Essential for bone mineralization and bone density	Low BMD, increased fracture risk
**Vitamin D**	Fatty fish (salmon, mackerel), fortified dairy products, eggs, mushrooms exposed to sunlight	Promotes calcium absorption in the intestines, supports bone mineralization	Poor calcium absorption, low BMD
**Protein**	Meat, fish, eggs, beans, legumes, dairy products	Supports muscle mass, which reduces fall risk; essential for bone matrix	Reduced bone mass, increased fracture risk
**Vitamin K**	Natto, leafy green vegetables (e.g., spinach, kale), broccoli	Assists in bone mineralization through osteocalcin activation	Reduced bone formation and quality
**Vitamin C**	Citrus fruits, bell peppers, strawberries, broccoli, tomatoes	Involved in collagen formation, essential for bone structure	Impaired bone formation, reduced bone strength
**Magnesium**	Nuts (e.g., almonds, cashews), leafy greens, whole grains, fish	Supports bone mineral density and calcium metabolism	Reduced bone density, increased fracture risk
**Phosphorus**	Meat, dairy, fish, eggs, nuts, legumes	Integral for bone mineral structure	Impaired bone mineralization
**Potassium**	Bananas, potatoes, oranges, spinach, tomatoes	May neutralize bone-depleting acids from high-protein diets	Increased risk of bone demineralization
**Zinc**	Meat, shellfish, legumes, nuts, seeds	Supports bone growth and maintenance	Impaired bone repair and density

BMD: bone mineral density.

## Abbreviations

OP	Osteoporosis
WHO	World Health Organization
BMD	Bone mineral density
T-score	Standard deviation score in BMD assessment
DEXA	Dual-emission X-ray absorptiometry
PTH	Parathyroid hormone
CKD	Chronic kidney disease
MBD	Mineral bone disorder
SHPT	Secondary hyperparathyroidism
RANKL	Receptor activator of nuclear factor kappa-B ligand
OPG	Osteoprotegerin
OC	Osteoclast
OB	Osteoblast
BPs	Bisphosphonates
GCs	Glucocorticoids
T2DM	Type 2 diabetes mellitus
ROS	Reactive oxygen species
AN	Anorexia nervosa
BN	Bulimia nervosa
MAT	Marrow adipose tissue
IGF-1	Insulin-like growth factor 1
GHD	Growth hormone deficiency
CD	Celiac disease
IL	Interleukin
IBD	Inflammatory bowel disease
HHC	Hemochromatosis
PBC	Primary biliary cholangitis
SERMs	Selective estrogen receptor modulators
RCT	Randomized control trial
SM	Systemic mastocytosis
BTM	Beta thalassemia major
RA	Rheumatoid arthritis
ACPAs	Anti-citrullinated protein antibodies
SLE	Systemic lupus erythematosus
OP	Osteoprotegerin
GnRH	Gonadotropin-releasing hormone
